# Contingency management is effective in promoting abstinence and retention in treatment among crack cocaine users with a previous history of poor treatment response: a crossover trial

**DOI:** 10.1186/s41155-019-0127-2

**Published:** 2019-07-15

**Authors:** André de Queiroz Constantino Miguel, Clarice Sandi Madruga, Viviane Simões, Rodolfo Yamauchi, Claudio Jerônimo da Silva, Michael McDonell, Sterling McPherson, John Roll, Ronaldo Ramos Laranjeira, Jair de Jesus Mari

**Affiliations:** 1 National Institute of Policies on Alcohol and Drugs (INPAD) of the Department of Psychiatry and Medical Psychology, Federal University of São Paulo (UNIFESP), Rua: Dr. Diogo de Faria, 1036, 3º Andar–Vila Clementino, São Paulo, SP Brazil; 2 Department of Psychiatry and Medical Psychology, Federal University of São Paulo (UNIFESP), Rua Borges Lagoa, 570 – 1o andar – Vila Clementino, São Paulo, SP 04038-030 Brazil; 3 Program of Excellence in Addictions Research, Washington State University, P.O Box 1469, Spokane, WA USA; 4 Spokane Valley, USA

**Keywords:** Crack cocaine, Contingency management, Behavior treatment, Psychosocial treatment, Brazil

## Abstract

**Background:**

Crack use has become a severe health problem in Brazil. Contingency management has shown robust evidence of efficacy in the treatment of cocaine use disorder (CUD) in high-income countries; however, it is still unclear how this intervention can impact treatment in low-income countries.

**Objective:**

To evaluate the efficacy of contingency management in the treatment of CUD among individuals with a previous history of poor treatment response in Brazil.

**Methods:**

Six months after the end of treatment, 32 participants previously allocated to the usual care condition (UCC) were invited to receive an additional 12 weeks of treatment in a contingency management condition (CMC), and 16 accepted the invitation. We compared data obtained from only the 16 participants (14 male) exposed to both treatment conditions.

**Results:**

Participants attended more treatment sessions and were retained in treatment for a longer period during the CMC than during the UCC (*p* < .01 for both). The proportion of negative cocaine samples submitted, the mean longest duration of cocaine abstinence, and the odds of being abstinent from cocaine during the 12 weeks of treatment were significantly higher during treatment in the CMC when compared to the UCC (*p* < .05).

**Conclusions:**

This study provides further evidence that contingency management is effective in promoting abstinence and retention in treatment among individuals with CUD with a history of poor treatment response. Our findings argue for the incorporation of CM among public treatment services for CUD in Brazil.

**Trial registration:**

This study was registered at ClinicalTrials.gov as NCT01815645 on March 21, 2013.

## Introduction

In the last two decades, crack cocaine use has risen at alarming rates in South America, but it has declined in Europe and the USA (Alessi, Hanson, Wieners, & Petry, 2007). In a recent study, 1.5% of Brazilians reported using crack cocaine in the past year (Abdalla et al., 2014), a rate 3 times higher than the mean of .6% reported for other South American countries (United Nations Office on Drugs and Crime, 2012). In Brazil, crack cocaine use is associated with concomitant severe psychiatric comorbidities such as alcohol use disorder, mood disorders, anxiety disorders, antisocial personality disorder, and suicidal ideation (Miguel et al., 2018; Narvaez et al., 2014a; Paim Kessler et al., 2012; Zubaran, Foresti, Thorell, Franceschini, & Homero, 2010). When compared with the general population, crack cocaine users present higher rates of unemployment, homelessness, exposure to violence, prostitution, and risky sexual behaviors, with elevated rates of sexually transmitted diseases such as HIV, hepatitis B, hepatitis C, and syphilis (Narvaez et al., 2014b; Pinto, Tancredi, Buchalla, & Miranda, 2014; Santos Cruz et al., 2013; Vernaglia, Vieira, & Cruz, 2015; von Diemen, De Boni, Kessler, Benzano, & Pechansky, 2010). Crack cocaine use is also associated with school dropout, illegal activities, incarceration, and mortality (Dunn & Laranjeira, 1999; Narvaez et al., 2015; Ribeiro, Dunn, Laranjeira, & Sesso, 2004; Ribeiro, Dunn, Sesso, Dias, & Laranjeira, 2006; Ribeiro, Sanchez, & Nappo, 2010; Santos Cruz et al., 2013).

Although the prevalence and profile of crack cocaine users in Brazil have been extensively studied over the past 20 years, little is known about the effectiveness of the existing treatments for cocaine use disorder (CUD) in the country. Contingency management (CM) interventions for substance use disorders have been applied in several countries such as the USA (Higgins et al., 1994; Petry et al., 2004), UK (Weaver et al., 2014), Spain (Garcia-Fernandez et al., 2011; Garcia-Rodriguez et al., 2009), and China (Chen et al., 2013). Several meta-analyses have indicated that CM is among the most effective psychosocial treatments in terms of retention in treatment, reduction of drug use, and promotion of continuous abstinence (Dutra et al., 2008; Lussier, Heil, Mongeon, Badger, & Higgins, 2006; Prendergast, Podus, Finney, Greenwell, & Roll, 2006). Likewise, CM is effective in the treatment of cocaine use disorder for patients who have poor family support or are homeless as well as for patients with poor response to previous CM treatment for substance use disorders (McDonell et al., 2013; Schumacher et al., 2007; Silverman, Chutuape, Bigelow, & Stitzer, 1999).

Recently, our group conducted the first randomized clinical trial (RCT) of CM for the treatment of CUD in Brazil (Miguel et al., 2016). We observed that, in comparison with the participants receiving usual care (control condition), those treated with CM had a lower frequency of cocaine use, achieved longer periods of continuous cocaine abstinence, and were more likely to be retained until the end of the treatment. CM was also more efficacious in reducing the frequency of marijuana and alcohol use, and psychiatric symptomatology (Miguel et al., 2016, 2017).

Although this first RCT contributed to the existing knowledge on CM and suggested CM’s efficacy in treating a Brazilian population with CUD, it is possible that these findings might have been influenced by the existence of one or more confounding variables unevenly distributed during the randomization process (e.g., distance from treatment facility, means of transportation, legal problems). In order to test this hypothesis and reinforce the reliability of our previous findings, we have conducted a crossover trial to determine the effects of CM on treatment outcomes protected from confounding factors related to sample bias. For this trial, participants randomized to the usual care condition in our original RCT (Miguel et al., 2016) were invited to receive the CM intervention 6 months after the end of treatment. The aim of this study was to compare how the same group of participants responded to treatment in terms of patient attendance, retention in treatment, and promotion of cocaine abstinence, when exposed to 12 weeks of usual care treatment and 12 weeks of usual care combined with CM treatment. Our hypothesis was that participants would show better response in all treatment outcomes when exposed to the CM treatment.

## Methods

### Study design

This was a crossover trial^1^ conducted with data obtained from a randomized controlled trial (RCT) that evaluated a contingency management intervention for CUD among individuals seeking treatment in the city of São Paulo, Brazil (Miguel et al., 2016). Six months after the end of treatment, participants previously randomized to 12 weeks of treatment in the usual care condition (UCC) were invited to receive an additional 12 weeks of treatment in the contingency management condition (CMC).

### Study site

The study was conducted at the Vila Maria Specialized Medical Outpatient Clinic for Alcohol and Drug Treatment (the referral clinic for the northern region of the city of São Paulo).

### Participants

During the first phase of this study (RCT design), a total of 86 individuals were screened for inclusion. The inclusion criteria were as follows: being between 18 and 60 years of age; seeking treatment for CUD; being currently dependent on cocaine according to the Diagnostic and Statistical Manual of Mental Disorders, fourth edition (DSM-IV) criteria (American Psychiatric Association, 1994); and crack cocaine being the primary drug of choice. Individuals who had been abstinent from crack cocaine for more than 4 weeks were excluded, as were those with a confirmed DSM-IV diagnosis of schizophrenia and those who were unable to attend treatment sessions at least three times per week. Of the 86 individuals screened, 21 did not meet the study criteria and were excluded. Therefore, for the first phase of this study, the sample was comprised of 65 individuals randomized to one of two treatment conditions: CMC for 12 weeks (*n* = 33) and UCC for 12 weeks (*n* = 32) (see Miguel et al., 2016 for full description of procedures and findings of this first phase of the study).

**Fig. 1:**
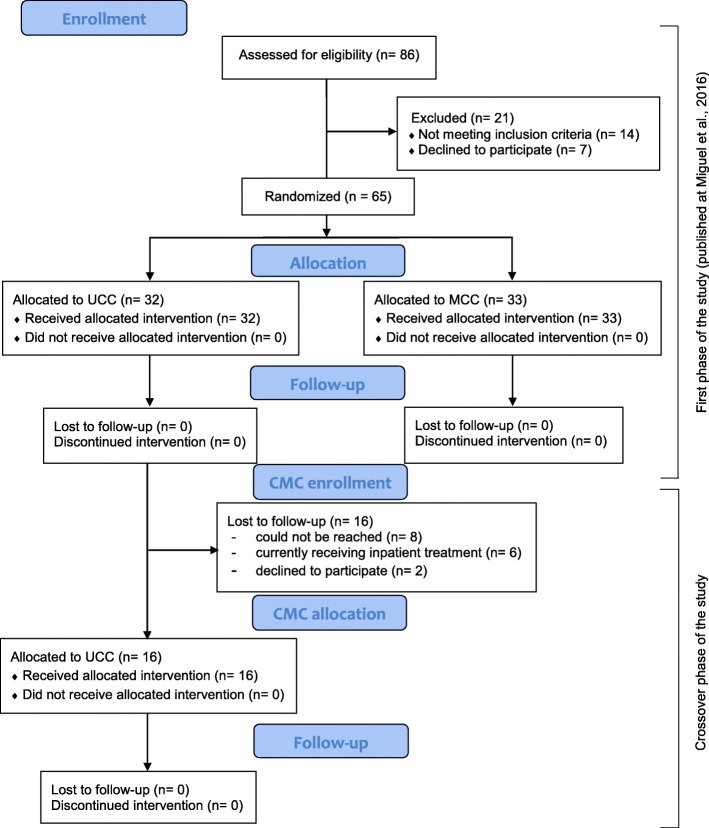
Flowchart of the study design. Flowchart of the study design according to the CONSORT diagram guidelines

For the second phase of the study (crossover design), participants in the UCC were encouraged to receive an additional 12 weeks of treatment in the CMC 6 months after the end of treatment in the UCC. Of the 32 participants who received treatment in the UCC in the initial phase of this trial, 16 participants (50%) were lost to follow-up (eight (25%) could not be reached, six (18.7%) were currently enrolled in an inpatient treatment program, and two (6.2%) declined the invitation to receive treatment in the CMC). Therefore, only 16 participants (50%) agreed to crossover from the UCC to the CMC. As a result, only the 16 participants that received both the UCC and CMC interventions were considered for our current analyses (Fig. 1).

All participants gave written informed consent. The study was approved by the Research Ethics Committee of the Federal University of São Paulo and by the Ethics Committee from the Brazilian National Ministry of Health (CAAE No: 00745912.4.0000.5505).

### Assessments

A questionnaire battery of baseline assessments was administered by trained research assistants at two different time points: 1 week prior to the UCC and 1 week prior to the CMC. Assessments included the collection of data related to sociodemographic characteristics, history/pattern of drug use, previous treatment history, and risky sexual behaviors. DSM-IV diagnoses of substance dependence and schizophrenia were assessed using the Structured Clinical Interview for DSM–IV Axis I Disorders (First, Spitzer, Gibbon, & Williams, 1997). The Brazilian Portuguese-language versions of the Beck Depressive Inventory II (Gorenstein, Pang, Argimon, & Werlang, 2011) and Beck Anxiety Inventory (Cunha, 2001) were also applied. After completing the assessments, participants submitted a urine sample (to assess recent cocaine use). Urine was sampled three times per week, and treatment session attendance was recorded.

### UCC

Participants in the UCC received the standard treatment provided by the Vila Maria Outpatient Clinic which consisted of one 90-min group therapy session focusing on relapse prevention and coping skills training per week, one 90-min group occupational therapy sessions per week, at least one individual session with a psychiatrist per month, and a maximum of one individual cognitive behavioral session focused on relapse prevention and coping skills training per week. Frequency of individual sessions was determined by treatment providers depending on patients’ needs. With the intent of maintaining treatment as usual, this variable was not controlled by researchers. A designated nurse case manager was responsible for monitoring, scheduling, and rescheduling participant visits as necessary. Participants were asked to submit urine samples three times per week (every Monday, Wednesday, and Friday). This procedure was coupled with a brief counseling (5 to 10 min) where participants were encouraged to stop using crack cocaine and remain in treatment. After submitting a sample, participants were immediately informed of the results; however, no form of monetary incentive was given to participants in the UCC group.

### CMC

Six months after the end of treatment, participants in the UCC were encouraged to initiate treatment in the CMC. For 12 weeks, participants received the exact same treatment as when they were in the UCC, together with the CM intervention. Usual care interventions were conducted by Vila Maria’s treatment staff while the contingency management intervention was conducted by the research staff. The contingency management intervention consisted of reinforcing cocaine abstinence by giving vouchers with monetary value after the submission of a urine sample that tested negative for cocaine. Urine samples were collected three times a week (every Monday, Wednesday, and Friday). Vouchers’ value started at ≅ US$1.25 (as of July 2018, one US dollar was equal to approximately four Brazilian reals) and increased by ≅ US$0.50 for every consecutive negative cocaine sample submitted to a maximum of ≅ US$5.00. An additional ≅ US$5.00 bonus was given if all three samples collected in a given week were negative for cocaine. Vouchers’ value was reset to the initial value if participants failed to submit a urine sample or were positive for cocaine. If abstinent from cocaine during the entire treatment period, participants would earn a total of ≅ US$225.00. Participants could exchange vouchers for goods immediately after submitting a urine sample that tested negative for cocaine. Vouchers could be exchanged for any goods available in the surrounding community (1.5-mile ratio of Vila Maria ambulatory), with the exception of tobacco and alcohol products. In order to exchange vouchers for goods, participants would go to a specific store to purchase a specific good accompanied by a research staff. Treatment staff would make the purchase using research money and deduct that value from the total amount earned by the study participant. Participants could also decide to save their vouchers without losing the accumulated value. However, vouchers could be exchanged for goods, only after submission of a cocaine-negative sample.

### Outcome measures

Treatment attendance was determined by the total number of sessions attended during the 12 weeks of treatment, and retention in treatment was determined considering the period elapsed between treatment intake and last appearance at the treatment facility.

Cocaine abstinence was accessed using five different cocaine use outcome measures recently associated with short- and long-term functional improvements (Carroll et al., 2014; Donovan et al., 2012; Kiluk et al., 2016; Miguel et al., 2019). These were proportion of cocaine-negative urine samples submitted (determined by dividing the total number of negative cocaine samples submitted by the total number of expected samples), longest duration of abstinence (LDA) (determined by the longest consecutive sequence of cocaine-negative samples submitted), achievement of three or more weeks of continuous abstinence (determined by the consecutive submission of nine or more cocaine-negative samples), retained and abstinent at the last week of treatment (determined by the presence in at least one treatment session and submission of all three cocaine-negative samples in the 12th week of treatment), and complete abstinent during treatment (determined by the submission of all 36 cocaine-negative urine samples).

### Statistical analysis

To assess if possible baseline characteristics or treatment outcome responses were associated with enrollment in the crossover phase of the trial, baseline assessments collected prior to the UCC and outcome responses collected at the end of the UCC intervention were compared for the 16 participants who crossed over to the CMC and the 16 participants who did not crossover (lost to follow-up or declined to participate). For these analyses, Fisher’s exact tests were used for dichotomous variables and *t* tests for independent samples were used for continuous variables.

In order to determine possible carryover effects, baseline assessments submitted 1 week prior to each treatment condition were compared for the 16 participants enrolled in the crossover phase of the trial. Due to the nature of our sample (paired), analyses were conducted using McNemar’s exact tests for dichotomous variables and paired *t* tests for continuous variables.

To assess the impact of the intervention on treatment attendance and retention in treatment, *t* tests for paired samples were conducted. To determine the impact of the intervention on cocaine abstinence, paired *t* tests were conducted with the proportion of cocaine-negative samples submitted and the longest duration of abstinence (in weeks). Additionally, three different McNemar exact tests were conducted considering the proportion of participants that were retained and abstinent at the last week of treatment, that achieved at least 3 weeks of continuous abstinence, and that were completely abstinent during treatment during each treatment condition.

Finally, a generalized estimation equation (GEE) for longitudinal binary outcomes was used to compare the development of both treatment conditions on cocaine use over time (36 assessments). Each assessment was coded as “1” for those subjects who came and submitted a negative urine exam and “0” for those who submitted a positive result. Holidays and being absent were coded as missing (holidays accounted for about 5% of the missing data). Due to the nature of the outcomes, a binary logistic model with an autoregressive structure of order 1 was built. For this model, six covariates were added: sex, age, alcohol dependence, baseline cocaine urine result, group condition, and time. Alcohol dependence and baseline urine results were included in this model following previous evidence that these variables are predictive of treatment response (Carroll, Rounsaville, & Bryant, 1993; Heil, Badger, & Higgins, 2001; Peirce et al., 2009; Stitzer et al., 2007). Sex and age were included for exploratory reasons only.

The alpha threshold of 0.05 was adopted in every model. *t* tests and exact tests were performed with the IBM SPSS Statistics software package, version 24.0 (IBM Corporation, Armonk, NY, USA) while the GEE models were conducted with StataSE 13 (StataCorp. 2013, College Station, TX: StataCorp LP).

### Dealing with missing data

To comply with the intention-to-treat paradigm, two imputation processes were adopted in order to evaluate the impact of missing data on the cocaine use outcome. First, all missing visits preceded and followed by negative samples were coded as negative. For our GEE analyses, no imputation was needed to achieve the intention-to-treat paradigm, and we used this estimate as a default. Finally, multiple imputations carried out via sequential regression imputation were also conducted for the GEE analyses. This latter approach fills in the data on a variable-by-variable basis, each time matching the imputation model to a variable’s distribution form (McPherson, Barbosa-Leiker, Burns, Howell, & Roll, 2012; McPherson, Barbosa-Leiker, McDonell, Howell, & Roll, 2013). Fifty imputed datasets were created and pooled to produce the GEE estimates (right-side). In modeling abstinence using GEE model, the missingness mechanism was considered as missing at random (MAR) because it is related to other measured variables in the analysis model (i.e., group assignment, the previous/posterior urine exam results) (McPherson et al., 2015). Furthermore, since we had mixed missing data causes (missing due to holiday and due to absence), the MAR assumption was used following previous evidence that it is the most adequate multiple imputation approach to return unbiased estimates (Baraldi & Enders, 2010; McPherson et al., 2012, 2013, 2015).

**Table 1:** Demographic characteristics, comorbid psychiatric disorders, history of drug use, and treatment outcome among participants who crossed and did not crossover

	Participants that crossed over to CMC (*n* = 16)		Participants that did not crossover to CMC (*n* = 16)		*p* value^1^
	*n* (%)	Mean ± SD	*n* (%)	Mean ± SD	
Baseline variables					
Age (years)		37.3 ± 8.4		33.9 ± 8.4	0.337
Male	14 (87.5)		14 (87.5)		1.000
Education (years of schooling)		10.1 ± 3.3		10 ± 4.2	0.612
Unemployment	11 (68.8)		16 (100)		0.043
Homelessness	3 (18.8)		6 (37.5)		0.433
Age at onset of crack use (years)		24.7 ± 8.4		23.2 ± 6.7	0.740
Duration of crack use (years)		12.6 ± 7.8		10.8 ± 7.3	0.444
Has slept on the streets due to crack use	6 (37.5)		12 (75.5)		0.073
Has been to Crackland^2^	14 (87.5)		16 (100)		0.484
History of inpatient treatment	14 (87.5)		12 (75.5)		1.000
Number of previous treatment attempts		4.6 ± 5.4		3.1 ± 3.1	0.282
Positive crack cocaine urine sample at baseline	9 (56.3)		7 (43.8)		0.654
Alcohol dependence	9 (56.3)		12 (75.5)		0.458
Multiple substance dependence	12 (75.0)		12 (75.0)		1.000
Psychotic symptoms	10 (62.5)		5 (31.2)		0.156
Beck Depression Inventory-II score		23.8 ± 12.6		19.9 ± 12.6	0.401
Beck Anxiety Inventory score		21.0 ± 14.0		10.6 ± 9.4	0.022
Treatment outcome variables					
Treatment attendance (sessions)		5.3 ± 7.7		2.3 ± 3.8	0.182
Retention in treatment (weeks)		3.9 ± 4.3		2.6 ± 3.7	0.489
Proportion of negative samples submitted		12.2 ± 19.9		5.7 ± 10.8	0.265
Longest duration of abstinence (weeks)		1.1 ± 2.2		0.5 ± 0.9	0.304
Retained and abstinent at the last week of treatment	0 (0)		0 (0)		1.000
3 or more weeks of abstinence	2 (12.5)		0 (0)		0.484
Completely abstinent during treatment	0 (0)		0 (0)		1.000

*CMC* contingency management condition, *SD* standard deviation

^1^*p* values were obtained using independent *t* test for continuous variables and Fisher’s exact test for dichotomous variables

^2^Crackland is a region of downtown São Paulo where thousands of people consume crack openly

## Results

### Sample characteristics prior to the interventions

As can be seen in Table 1, at the time of intake to the first phase of the study, baseline characteristics were similar overall for participants who crossed over to CMC compared to participants who did not crossover. For the variables that achieved statistical differences, the proportion of unemployment at treatment intake was higher among those that did not crossover to the CMC (68.8% vs. 100%; *p* < .05), while the mean Beck Anxiety Inventory score was higher among those that did crossover (21.0 ± 14.0 vs. 10.6 ± 9.4; *p* < .05). Treatment outcome responses did not differ statistically between those who crossed and did not crossover to the CMC.

**Table 2:** Demographic characteristics, comorbid psychiatric disorders, and patterns of drug use among crack cocaine users in the city of São Paulo, Brazil, prior to treatment intervention

Variable	Prior to UC intervention (*n* = 16)		Prior to CM intervention (*n* = 16)		*p* value^1^
	*n* (%)	Mean ± SD	*n* (%)	Mean ± SD	
Age (years)		37.3 ± 8.4		37.9 ± 8.7	0.838
Male	14 (87.5)		14 (87.5)		1.000
Education (years of schooling)		10.1 ± 3.3		9.3 ± 3.3	0.590
Unemployment	11 (68.8)		13 (81.2)		0.500
Homelessness	3 (18.8)		4 (25.0)		1.000
Age at onset of crack use (years)		24.7 ± 8.4		23.9 ± 8.6	0.777
Duration of crack use (years)		12.6 ± 7.8		14 ± 6.8	0.542
Has slept on the streets due to crack use	6 (37.5)		8 (50.0)		0.250
Has been to Crackland*	14 (87.5)		12 (75.5)		0.500
History of inpatient treatment	14 (87.5)		14 (87.5)		1.000
Number of previous treatment attempts		4.6 ± 5.4		4.8 ± 4.1	0.918
Positive crack cocaine urine sample at baseline	9 (56.3)		6 (37.5)		0.727
Alcohol dependence	9 (56.3)		9 (56.3)		1.000
Multiple substance dependence	12 (75.0)		12 (75.0)		1.000
Psychotic symptoms, *n* (%)	10 (62.5)		10 (62.5)		1.000
Beck Depression Inventory-II score		23.8 ± 12.6		20.3 ± 12.1	0.493
Beck Anxiety Inventory score		21.0 ± 14.0		23.1 ± 11.9	0.694

*UC* usual care, *CM* contingency management, *SD* standard deviation

^*^Crackland is a region of downtown São Paulo where thousands of people consume crack openly

^1^*p* values were obtained using paired *t* test for continuous variables and the McNemar exact test for dichotomous variables

Participants’ pretreatment characteristics obtained 1 week prior to both interventions are compared in Table 2. The studied sample was composed predominantly of men (over 85%), with a low level of education and high rates of unemployment (around 70%). Nearly a quarter of the participants reported living on the streets. The mean age at crack cocaine use onset was just over 23 years, and the mean duration of continuous crack cocaine use was 12 years. Approximately half of the participants reported sleeping on the streets due to crack cocaine use, and more than 75% reported spending their time in “Crackland”^2^ in order to use crack. Regarding other psychiatric disorders, the prevalence of current multiple substance dependence was extremely high (75%), with over half of the participants meeting DSM-IV criteria for current alcohol dependence. More than 60% reported having had at least one psychotic episode, with mean depressive and anxiety symptomatology scores in the moderate range. Most participants (over 80%) had a history of inpatient treatment for CUD. Demographic characteristics, patterns of drug use, prevalence of other substance use disorders, and psychiatric symptomatology did not differ statistically between the assessments made prior to the UCC and CMC interventions.

**Table 3:** Attendance, retention, and crack cocaine abstinence outcomes by treatment condition

Variable	During UC intervention (*n* = 16)		During to CM intervention (*n* = 16)		*t*/OR	*p* value^1^
	*n* (%)	Mean ± SD	*n* (%)	Mean ± SD		
Treatment attendance (sessions)		5.3 ± 7.9		19.9 ± 15.4	4.02	0.001
Retention in treatment (weeks)		3.9 ± 4.2		9.5 ± 3.3	4.89	0.001
Proportion of negative samples submitted		12.2 ± 19.9		45.3 ± 40.9	3.21	0.005
Longest duration of abstinence (weeks)		3.4 ± 6.4		12.6 ± 14.7	2.29	0.047
Retained and abstinent at the last week of treatment	0 (0)		7 (43.5)		–	0.016
3 or more weeks of abstinence	2 (12.5)		7 (43.5)		6	0.125
Completely abstinent during treatment	0 (0)		4 (25)		–	0.125

*UC* usual care, *CM* contingency management, *SD* standard deviation

^1^*p* values were obtained using paired *t* test for continuous variables and the McNemar exact test for dichotomous variables

### Treatment outcome responses

As seen in Table 3, during the UCC, participants attended a mean of 5.3 ± 7.9 treatment sessions, in comparison to 19.9 ± 15.4 sessions attended during the CMC. The difference between these two conditions was statistically significant (*t =* 4.02; *p* < .01). Similarly, the mean number of weeks retained in treatment was significantly lower in the UCC in comparison to the CMC (3.9 ± 4.2 versus 9.5 ± 3.3; *t =* 4.89; *p* < .01. The proportion of participants testing negative for cocaine was significantly higher during the CMC in comparison to the UCC when all expected urine samples were considered (45.3% vs. 12.2%; *t =* 3.21; *p* < .01). In regard to the longest duration of abstinence, the mean longest consecutive number of cocaine-negative urine samples submitted was 3.4 ± 6.4 in the UCC and 12.6 ± 14.7 in the CMC, corresponding to a mean of 1.1 ± 2.2 vs. 4.2 ± 4.9 weeks of continuous cocaine abstinence, respectively. This difference was also statistically significant (*t* = 2.23; *p* < .05). The proportion of participants retained and abstinent at the last week of treatment was significantly higher in the CMC than in UCC (43.8% vs. 0; *p* < .05). Similarly, the proportion of participants achieving 3 or more weeks of abstinence (43.8% vs. 12.5%) or being completely abstinent during treatment (25% vs. 0%) was higher during the CMC; however, these differences were only observed in a trending level (*p* = 0.125 for both).

As can be seen in Table 4, the GEE default abstinence results showed a statistically significant effect of the treatment condition (OR = 4.87; *p* < .01). In other words, the chance of being abstinent at any given point of the 12-week intervention was five times higher during the CMC when compared to the UCC. Such an outcome is consistent with the imputed GEE results that indicated that the chances of submitting a negative cocaine urine result were over 7 times higher under the CMC condition (OR = 7.39; *p <* .01). For the default results, no significant differences were observed for the covariates. Nonetheless, the imputed data led to significant statistical differences for the covariates alcohol dependence, baseline urine result, and time. Regardless of treatment condition, participants with alcohol dependence had .27 less chance of being abstinent from cocaine at any given point than those without an alcohol dependence diagnosis (*p <* .05), while participants that were negative for cocaine at baseline had 2.4 times higher chance of being abstinent at any given time when compared to those positive for cocaine at baseline (*p <* .05). Finally, the odds of being negative for cocaine reduced by a .98 ratio after every treatment assessment independent of treatment condition (*p < *.05). No significant differences were observed for the covariates age and gender (*p >* .35 for both).

**Table 4:** Abstinence outcome over time among crack dependent individuals

	Default				Multiple imputation (pooled datasets)	
	OR*	95% CI	SE	*p* value	OR*	*p* value
Treatment condition	4.87	1.56–15.21	2.83	0.006	7.39	0.003
Age	1.01	0.93–1.09	0.04	0.79	1.02	0.35
Alcohol dependence	0.48	0.12–1.98	0.35	0.31	0.27	0.02
Gender	1.38	0.19–9.87	1.38	0.75	1.41	0.65
Baseline urine result	1.12	0.38–3.30	0.62	0.84	2.37	0.01
Time	0.98	0.95–1.00	0.01	0.33	0.96	0.01

*The reference was the usual care condition. *OR* odds ratio, *CI* confidence interval

## Discussion

Crack cocaine use has become a major health problem in Brazil, where public treatment facilities still struggle to develop effective interventions to treat this population. CM interventions have proven effective in the treatment of CUD in a variety of contexts (Dutra et al., 2008; Lussier et al., 2006; Prendergast et al., 2006; Schumacher et al., 2007) and have recently shown efficacy in the treatment of CUD in Brazil (Miguel et al., 2016, 2017, 2018). The present crossover study was developed to further assess the efficacy of CM in Brazil by comparing how same-group participants with CUD respond to CM in comparison to usual care.

Our sample was composed predominantly of men, with over 10 years of continuous crack cocaine use, a low level of education, and high rates of unemployment and homelessness. There was also a high prevalence of concomitant psychiatric symptomatology and other substance use disorders. These characteristics are aligned with the findings of previous observational studies of this population (Narvaez et al., 2014a, 2015; Paim Kessler et al., 2012; Santos Cruz et al., 2013) and demonstrate the critical state of health and social conditions among crack cocaine users in Brazil. Furthermore, the fact that most participants had several previous treatment attempts (mean of 4.8) with the great majority of them having at least one inpatient treatment experience (87%) points to the lack of efficacy of the existing Brazilian interventions in treating this extremely severe population.

Of note is the fact that intake assessments collected prior to each treatment condition showed no statistical difference for any variable and thus suggests the homogeneity of the sample during those two different time points, and argues for the lack of carryover effects derived from the usual care intervention. As a result, any difference in within treatment outcomes seen between the CMC and the UCC is likely to be the result of the effects of each intervention.

Poor attendance and dropout are common in outpatient treatments for CUD and are associated with relapse and continued drug use (Ball, Carroll, Canning-Ball, & Rounsaville, 2006). In the present study, we observed that participants attended significantly more treatment sessions and were retained in treatment for a longer period when they received CM compared to the period receiving usual care treatment. These findings are aligned with previous evidence of the efficacy of contingency management in promoting attendance and retention in treatment among crack cocaine users in Brazil (Miguel et al., 2016) and other countries (Garcia-Fernandez et al., 2011; Garcia-Rodriguez et al., 2009; Higgins et al., 1994; Petry et al., 2004).

The achievement of sustained periods of abstinence is broadly accepted among the most desirable treatment outcomes and is currently the only outcome accepted by the US Food and Drug Administration (FDA) for cocaine use disorder treatments (Donovan et al., 2012; Food and Drug Administration and Psychopharmacologic Drugs Advisory Committee, 2013). In addition to sustained abstinence, other cocaine-related outcome measures have shown to be associated with significant long-term functional improvements warranting their use in treatment trials for CUD (Carroll et al., 2014; Donovan et al., 2012; Morean et al., 2015).

With this as a reference, in this crossover trial, we evaluated cocaine abstinence using 5 different outcome measures. The poor performance observed in the UCC in all cocaine abstinence outcome measures provides additional evidence of the inefficacy of the existing treatments for CUD in Brazil. On the other hand, the improvements observed during the CMC point towards the efficacy of CM in promoting cocaine abstinence in Brazil. These findings are consistent with those of a previous CM trial involving crack cocaine users in Brazil (Miguel et al., 2016), as well as with CM studies involving cocaine users conducted in the USA (Higgins et al., 1994; Petry et al., 2004) and Spain (Garcia-Fernandez et al., 2011; Garcia-Rodriguez et al., 2009). Finally, our findings argue for the efficacy of CM in promoting cocaine abstinence for severe crack cocaine users with a history of unsuccessful treatment attempts.

### Limitations

It is important to consider these results within the limitations of our study. First, several participants were lost to follow-up during the UCC. As a result, only 50% of the participants that received treatment in the UCC were enrolled to receive treatment in the CMC. Therefore, our results might have been different if all 32 subjects were enrolled in this study. However, the fact that baseline characteristics and within treatment outcomes were similar for UCC participants who crossed over compared to those that did not crossover reduces the chances of attritional bias due to follow-up losses. Second, there was a substantial number of missing urine samples, especially in the UCC. This could be due, in part, to the lack of efficacy of the UCC and the severe health and social conditions of the participants in our sample, as well as the fact that participants did not receive any form of incentive to submit urine samples during the UCC. Nevertheless, this phenomenon restricted our access to the actual data, thus limiting the accuracy of our findings. To deal with missing urine samples, we used data deletion (considering missing as missing) and multiple imputation. Although both of these approaches have been used extensively in outpatient clinical trials for substance use disorders, either can produce biased results. Hence, it is important to acknowledge that different results (including smaller differences between treatment conditions) might have been obtained if there had been more data available for analysis from the UCC. Third, our study had a relatively small sample rendering it underpowered to uncover differences with smaller effect sizes. It is possible that outcome differences observed here in only trending levels might have been confirmed within a larger sample. On the other hand, crossover trials are considered to be statistically efficient and, since participants serve as their own control, are protected from possible confounding factors related to the randomization process. Hence, the methodological qualities of a crossover design might have, in part, reduced our sample size limitation. Four, this study included only outcomes accessed during treatment enabling us to only compare the short-term effects of these treatment interventions.

Lastly, our study was conducted at a single facility with a particular treatment protocol for a specific crack cocaine-using population. Therefore, it is difficult to determine to what extent our findings can be generalized, if we consider the distinctive characteristics of crack cocaine users, as well as the many different treatment settings and protocols currently employed in Brazil.

## Conclusion

The present study provides further evidence of the efficacy of CM in promoting attendance, retention, and cocaine abstinence among crack cocaine users with a recent history of poor treatment response to usual care interventions. Our findings support the incorporation of CM as an additional treatment component to public treatment services for CUD in Brazil. The implementation of CM in outpatient treatment settings for crack cocaine use can be an effective strategy to address this public health scourge, and therefore, the dissemination of CM to researchers, health providers, and policymakers should be encouraged. Further studies, involving larger samples, conducted at multiple centers and designed to address effectiveness, as well as cost-effectiveness, are warranted.

## Data Availability

Please contact the first author for data requests.

## Abbreviations

BAI: Beck Anxiety Inventory
BDI: Beck Depressive Inventory
CM: Contingency management
CMC: Contingency management condition
GEE: General estimating equation
MAR: Missing at random
UCC: Usual care condition

## Footnotes

1 Crossover trials are longitudinal controlled studies where a same group of participants are exposed to two or more different interventions. By doing so, same participants serve as their own control.

2 “Crackland” is a region of downtown São Paulo where thousands of crack users living under extremely vulnerable social conditions gather to consume crack openly in the streets.
